# Supplementary material for: Photocured room temperature phosphorescent materials from lignosulfonate
Source: Nat Commun. 2024 Feb 21;15:1590. doi: 10.1038/s41467-024-45622-3 (PMC10881523; doi:10.1038/s41467-024-45622-3)
Supplement: Supplementary file 1 — Supplementary Information [file 41467_2024_45622_MOESM1_ESM.pdf]

Supplementary information

Materials

Lignosulfonate (average M_w ~52,000) and kraft lignin (average M_w ~10,000) were purchased from Sigma-Aldrich (Shanghai, China). Alkali lignin was purchased from Aladdin (Shanghai, China). Enzymatic hydrolysis lignin was purchased from Longlive Biological Technology Co.Ltd. (Shandong, China). Acrylamide (99.0%), acrylic acid (>99.7%), methyl acrylate (≥99.5%), methyl methacrylate (>99.5%), styrene (>99.5%), N-isopropylacrylamide (98%) were purchased from Aladdin (Shanghai, China). 2-Hydroxy-4'-(2-hydroxyethoxy)-2-methylpropiophenone (>98.0%) and benzophenone (99.5%) were purchased from Aladdin (Shanghai, China). Ionic liquids [1-ethyl-3-methylimidazolium bromide (>98%), 1-ethyl-3-methylimidazolium chloride (>98%), 1-ethyl-3-methylimidazolium acetate (>97%)] were purchased from Aladdin (Shanghai, China). 1,4-dioxane (99.5%), acetonitrile (99.5%), ethyl alcohol (99.5%), petroleum ether (99.5%), N,N-dimethylformamide (99.5%), tetrahydrofuran (99.5%), dichloromethane (99.5%), ethyl acetate (99.5%), methylbenzene (99.5%), acetone (99.5%) were purchased from Aladdin (Shanghai, China). Rhodamine B (>99.75%) was purchased from Kermel Chemical Industry (Tianjin, China). Radical scavenger 5,5-dimethyl-1-pyrroline N-oxide (97%) was purchased from Aladdin (Shanghai, China). Thermal initiator ammonium persulfate (>99.0%) was purchased from Macklin (Shanghai, China). Poly (vinyl alcohol) (DP=1750±50, 97%) was purchased from Macklin (Shanghai, China). Deionized water was produced using a Smart-RO ultrapure water system (Hitech Instruments Co., Ltd., Shanghai, China). LED-UV curing lamp (photocuring wavelength =365nm, optical density =170mW/cm^{-2}) was purchased form Zhongshan UV curing lighting appliance factory in Taobao (Alibaba, Hangzhou, China). All molds made of silicone and screen printing molds were customized from Chinese cabbage screen printing equipment main shop in Taobao (Alibaba, Hangzhou, China).

Characterization

About 100 mg of lignosulfonate was dissolved in 0.5 mL of D_2O for 2D-HSQC spectra accumulation. The Bruker standard pulse program "hsqcetgp" was used for

HSQC experiments. The spectral widths were 5000 Hz and 20000 Hz for the ^{1}H- and ^{13}C-dimensions, respectively. The number of collected complex points was 1024 for ^{1}H-dimension with a recycle delay of 1.5 s. The number of transients was 32, and 256-time increments were always recorded in the ^{13}C-dimension. The 1JCH used was 145 Hz. Prior to Fourier transformation, the data matrixes were zero filled up to 1024 points in the ^{13}C-dimension. Data processing was performed using standard Bruker Topspin-NMR software. UV-Vis absorption spectra were recorded using a TU-1901 UV-Vis double-beam spectrophotometer (Persee General Instrument Co., Ltd., Beijing, China). The light transmittance of samples were measured using a Lambda 950 (Varian) with a wavelength range of 370–800 nm. Fluorescence spectra, phosphorescence spectra and lifetime decay curves were recorded using an FLS1000 photoluminescence spectrometer (Edinburgh Instruments, Livingston, UK), equipped with a xenon lamp and a microsecond flashlamp (detector: photomultiplier tube, 200 nm $< \lambda <$ 1700 nm). Afterglow emission spectra were recorded after a 10ms delay. The temperature was controlled using an OX135QX cryostat (Oxford Instruments plc, Abingdon, UK). Fourier transform-infrared (FT-IR) spectra were recorded using a Fourier FT-IR spectrometer (Perkin Elmer Inc., Waltham, MA, USA). In situ FT-IR was measured using a Thermo Scientific Nicolet iS20. ESR analysis and high-field ESR analysis was performed on a Bruker EMXplus-6/1 spectrometer with a center field at 3500 G and a sweep width of 100 G at room temperature. Before conducting the above characterization, the samples were dried in an oven to prevent moisture from affecting the experimental results. All photos and videos were taken using a mobile phone.

Supplementary methods

Photocuring of P-AA

A mixture of acrylic acid (500 mg, 6.94 mmol), lignosulfonate (1 mg) and 1-ethyl-3-methylimidazolium bromide (400 mg, 2.09 mmol) were heated at 80 °C until the mixture changed to a homogeneous solution. After that, the solution was exposed to UV LED light sources (365 nm, 170 mW cm^{-2}) for 20 min for photocuring.

Photocuring of P-MA

A mixture of methyl acrylate (400mg, 4.65 mmol), acrylic acid (100 mg, 1.39 mmol), lignosulfonate (1 mg) and 1-ethyl-3-methylimidazolium bromide (400 mg, 2.09 mmol) were heated at 80 °C until the mixture changed to a homogeneous solution. After that, the solution was exposed to UV LED light sources (365 nm, 170 mW cm^{-2}) for 20 min for photocuring.

Photocuring of P-MMA

A mixture of methyl methacrylate (400mg, 3.99 mmol), acrylic acid (100 mg, 1.39 mmol), lignosulfonate (1 mg) and 1-ethyl-3-methylimidazolium bromide (400 mg, 2.09 mmol) were heated at 80 °C until the mixture changed to a homogeneous solution. After that, the solution was exposed to UV LED light sources (365 nm, 170 mW cm^{-2}) for 20 min for photocuring.

Photocuring of P-SM

A mixture of styrene (400mg, 3.84 mmol), acrylic acid (100 mg, 1.39 mmol), lignosulfonate (1 mg) and 1-ethyl-3-methylimidazolium bromide (400 mg, 2.09 mmol) were heated at 80 °C until the mixture changed to a homogeneous solution. After that, the solution was exposed to UV LED light sources (365 nm, 170 mW cm^{-2}) for 20 min for photocuring.

Photocuring of P-NIPAM

A mixture of N-isopropylacrylamide (500 mg, 4.42 mmol), lignosulfonate (1 mg) and 1-ethyl-3-methylimidazolium bromide (400 mg, 2.09 mmol) were heated at 80 °C until the mixture changed to a homogeneous solution. After that, the solution was exposed to UV LED light sources (365 nm, 170 mW cm^{-2}) for 20 min for photocuring.

Preparation of Ir2959-P-Lig

A mixture of acrylamide (500 mg, 7.03 mmol), lignosulfonate (1 mg), and 1-ethyl-3-methylimidazolium bromide (400 mg, 2.09 mmol) were heated at 80 °C until the mixture changed to a homogeneous solution. After that, 2-Hydroxy-4'-(2-hydroxyethoxy)-2-methylpropiophenone (1mg, 4.46 μmol) was add to the above solution, and then the solution was exposed to UV LED light sources (365 nm, 170 mW cm^{-2}) for 20 min for photocuring.

Preparation of BP-P-Lig

A mixture of acrylamide (500 mg, 7.03 mmol), lignosulfonate (1 mg), and 1-ethyl-3-methylimidazolium bromide (400 mg, 2.09 mmol) were heated at 80 °C until the mixture changed to a homogeneous solution. After that, benzophenone (1mg, 5.49 μmol) was add to the above solution, and then the solution was exposed to UV LED light sources (365 nm, 170 mW cm^{-2}) for 20 min for photocuring.

Preparation of P-BpA

A mixture of acrylamide (500 mg, 7.03 mmol), ammonium persulfate (1 mg), phenylboronic acid (1 mg, 8.2 μmol) and 1-ethyl-3-methylimidazolium bromide (400 mg, 2.09 mmol) were heated at 60 °C for 30 min. After that, a cured control sample was obtained for measurement

Preparation of Ir2959-P-BpA

A mixture of acrylamide (500 mg, 7.03 mmol), phenylboronic acid (1 mg, 8.2 μmol) and 1-ethyl-3-methylimidazolium bromide (400 mg, 2.09 mmol) were heated at 80 °C until the mixture changed to a homogeneous solution. After that, 2-Hydroxy-4'-(2-hydroxyethoxy)-2-methylpropiophenone (1mg, 4.46 μmol) was add to the above solution, and then the solution was exposed to UV LED light sources (365 nm, 170 mW cm^{-2}) for 20 min for photocuring.

Preparation of BP-P-BpA

A mixture of acrylamide (500 mg, 7.03 mmol), phenylboronic acid (1 mg, 8.2 μmol) and 1-ethyl-3-methylimidazolium bromide (400 mg, 2.09 mmol) were heated at 80 °C until the mixture changed to a homogeneous solution. After that, benzophenone (1mg, 5.49 μmol) was add to the above solution, and then the solution was exposed to UV LED light sources (365 nm, 170 mW cm^{-2}) for 20 min for photocuring.

Preparation of P-AM

A mixture of acrylamide (500 mg, 7.03 mmol), ammonium persulfate (1 mg), and 1-ethyl-3-methylimidazolium bromide (400 mg, 2.09 mmol) were heated at 60 °C for 30 min. After that, a cured control sample was obtained for measurement

Preparation of Ir2959-P-AM

A mixture of acrylamide (500 mg, 7.03 mmol), and 1-ethyl-3-methylimidazolium bromide (400 mg, 2.09 mmol) were heated at 80 ℃ until the mixture changed to a homogeneous solution. After that, 2-Hydroxy-4'-(2-hydroxyethoxy)-2-methylpropiophenone (1 mg, 4.46 μmol) was add to the above solution, and then the solution was exposed to UV LED light sources (365 nm, 170 mW cm^{-2}) for 20 min for photocuring.

Preparation of photocured materials with different lignin

The method for preparation of P-Kraft, P-AL and P-EL is as same as the photocuring of P-Lig, just need to replace lignosulfonate with kraft lignin, alkali lignin and enzymatic hydrolysis lignin of the same quality.

Recyclability of the ionic liquid

The P-Lig was dissolved in the water and was treated using dialysis bag for 5 h. The water solution obtained outside the dialysis bag was washed using ethyl acetate. Then, the recycled ionic liquid was obtained by evaporating the water.

^{1}H NMR analysis experiment

To detect the interaction of lignosulfonate with the aforementioned components, a mixture of acrylamide (500 mg, 7.03 mmol), lignosulfonate (20 mg) and 1-ethyl-3-methylimidazolium bromide (400 mg, 2.09 mmol) were heated at 80 ℃ until the mixture changed to a homogeneous solution; After that, the solution was exposed to UV LED light sources (365 nm, 170 mW cm^{-2}) for 20 min for photocuring; And then, the ionic liquid was removed by dialysis for 5 h; Finally, the remaining part was dissolved in D_2O to perform ^{1}H NMR.

Simulation methods

1 Determination of the conformation of the complex

Use MOLCLUS software to generate the initial guessed conformation of bimolecular complex, then call MOPAC [2] to calculate using PM6-D3H4 algorithm[1], take the conformation with the lowest energy as the most stable conformation, use ORCA[2] software for geometric optimization and single-point energy calculation, the parameters are set as follows, use density functional method, B3LYP exchange correlation functional, def2-TZVP basis set for calculation, in order to accurately describe the

dispersion effect, use D3(BJ) dispersion Correction[3]. Using the SMD solvent model[4], the solvent is water.

2 Binding energy calculation

The formula (1) for calculating the binding energy is:

$$E_{binding\ energy} = E_{total} - E_{mol1} - E_{mol2} \quad (1)$$

E_{total} is the single-point energy of the bimolecular complex structure, and E_{mol1} and E_{mol2} are the single-point energies of the two molecules involved in the complex, respectively.

3 RDG function

In order to investigate the weak interaction of monomers in the complex, including van der Waals interaction, hydrogen bond interaction, etc., the RDG function[5] analysis is carried out, and the RDG function isosurface diagram is drawn[6]. The RDG function (2) is defined as follows:

$$\mathrm{RDG}(r) = \frac{1}{2(3\pi^2)^{1/3}} \frac{|\nabla\rho(r)|}{\rho(r)^{4/3}} \quad (2)$$

where ρ is the electron density and $\nabla\rho$ is the electron density derivative. From the analysis results, the region and strength of the weak interaction can be known.

The colors on the RDG isosurface represent different types of interactions, among which blue represents hydrogen bonds and strong halogen bonds, etc., green represents van der Waals interactions, and red represents strong mutual repulsion, such as steric effects in rings and cages.

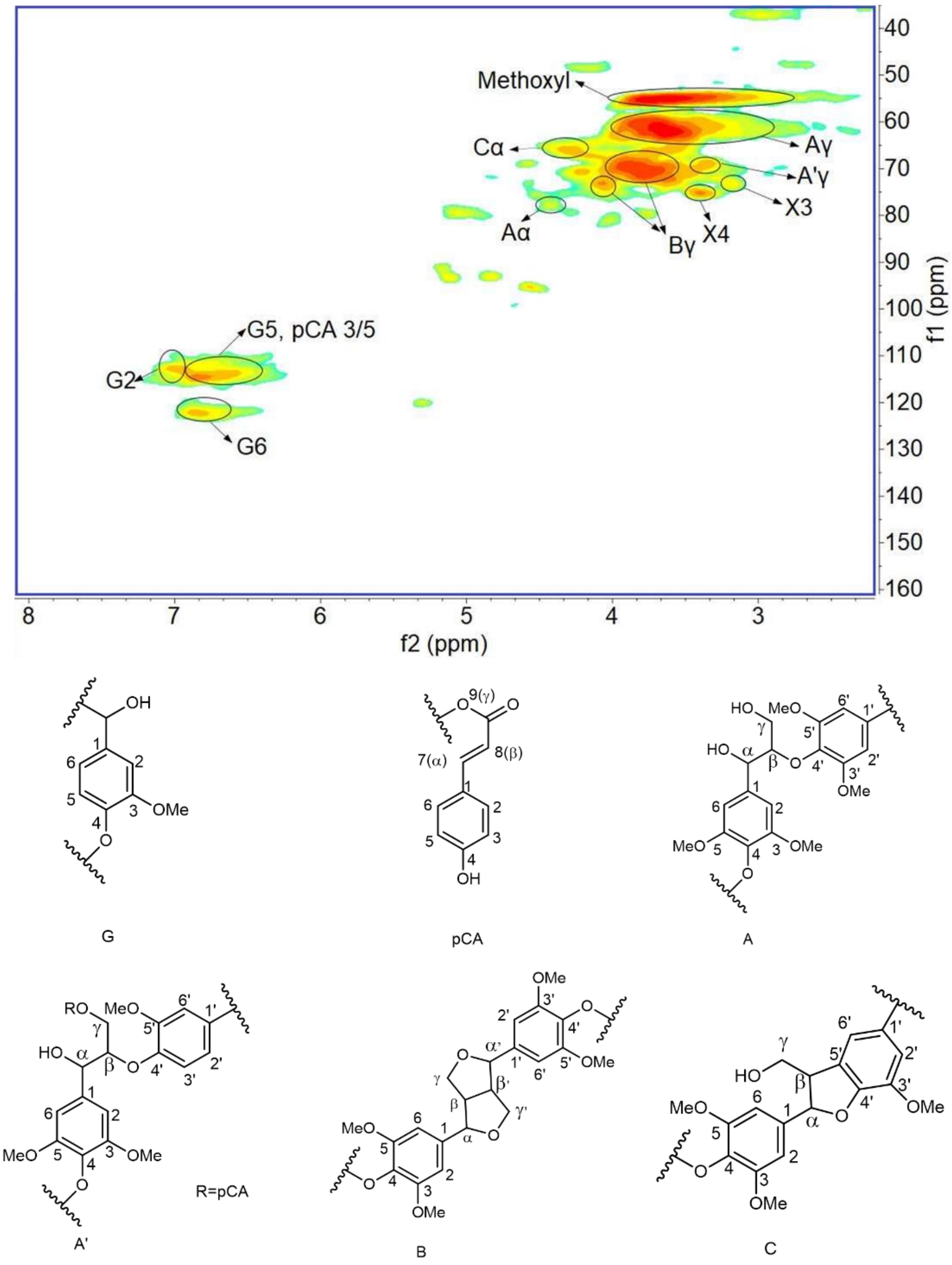

Supplementary Fig. 1 2D HSQC NMR spectra of lignosulfonate (X represents xylan).

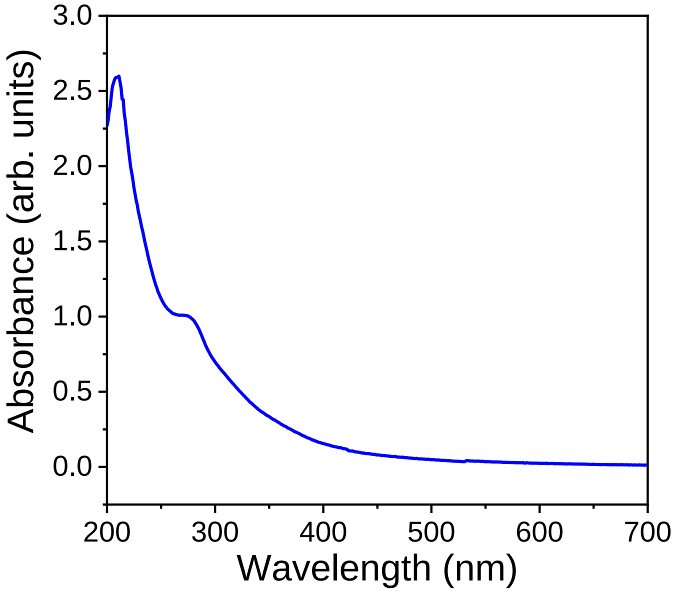

Supplementary Fig. 2 UV-Vis spectra of lignosulfonate in water (0.02 mg/ml).

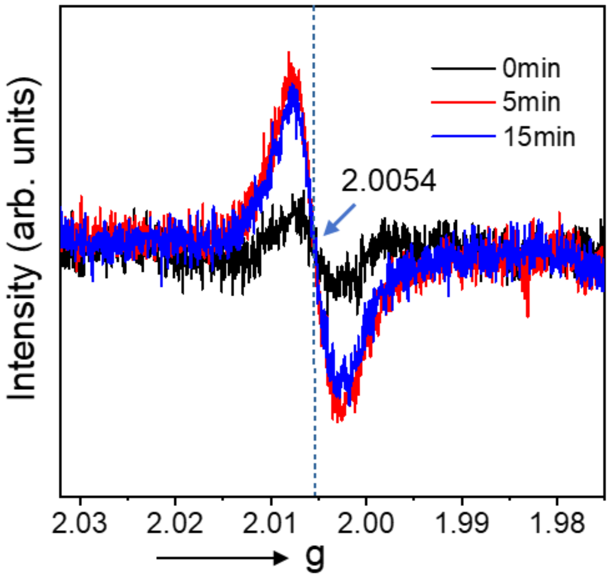

Supplementary Fig. 3 ESR spectra of lignosulfonate in ionic liquid upon UV irradiation for o min (black line), 5 min (red line), and 15min (blue line), g factor is 2.0054.

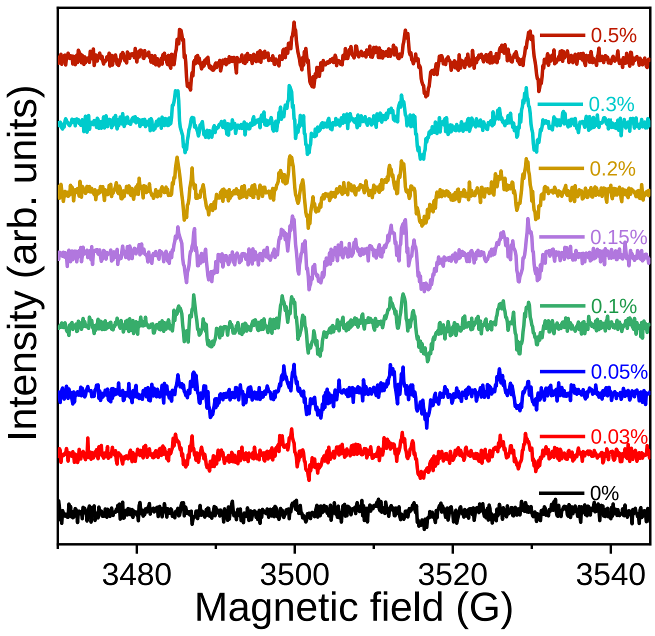

Supplementary Fig. 4 ESR spectra of different concentrations (mass percent) of lignosulfonate dissolved in ionic liquids upon UV irradiation for 10 min.

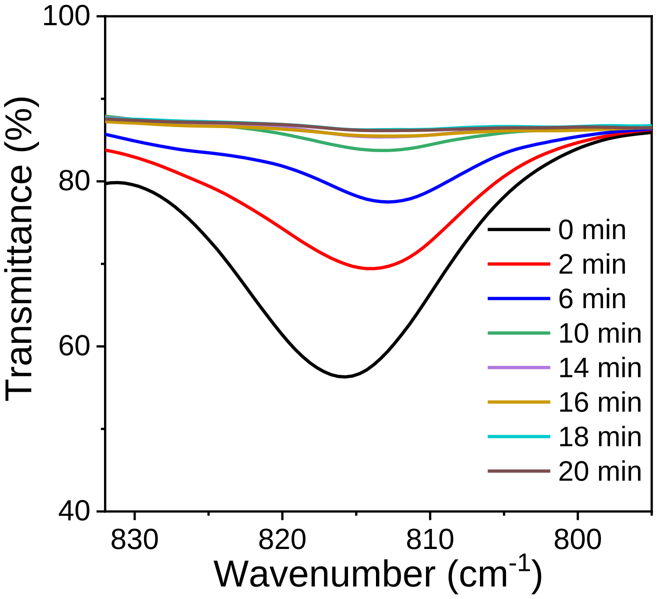

Supplementary Fig. 5 FT-IR spectra of P-Lig upon UV irradiation for different times.

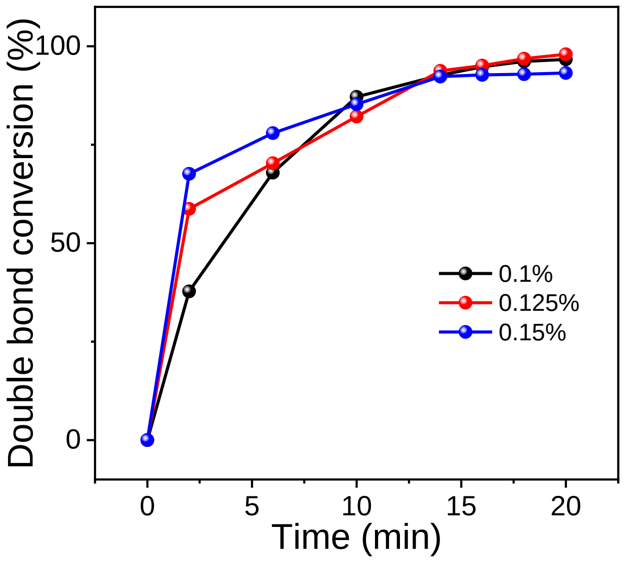

Supplementary Fig. 6 Double bond conversion of P-Lig prepared with different concentrations (mass percent) of lignosulfonate (black line represents 0.1% w/w, red line represents 0.125% w/w and blue line represents 0.15% w/w).

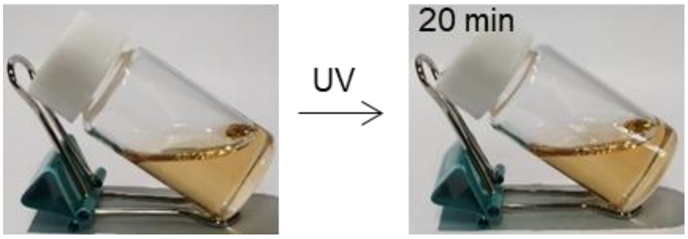

Supplementary Fig. 7 Digital images of the photocuring process of P-Lig after adding DMPO (100 μM).

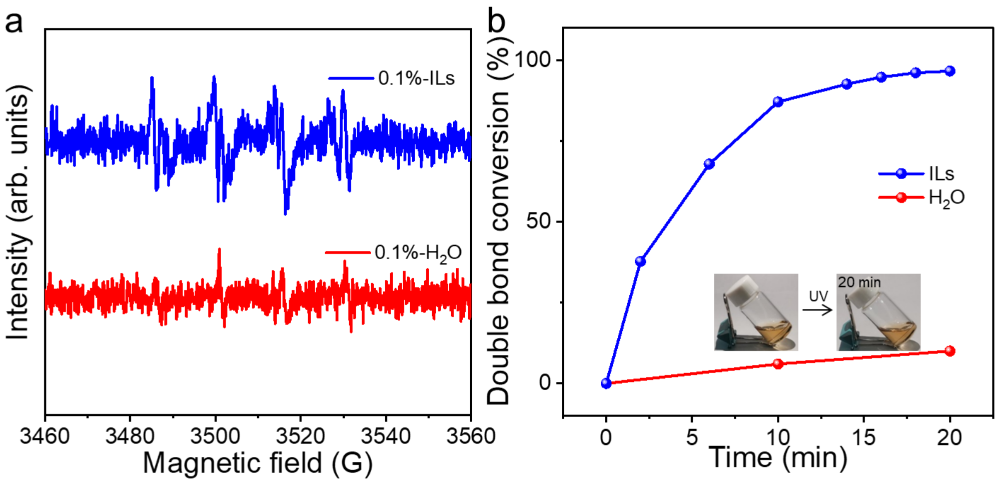

Supplementary Fig. 8 Photoradicals of lignosulfonate and photocuring process of P-Lig in water and ionic liquid. a) ESR spectra of lignosulfonate (0.1% w/w) in ionic

liquid (blue line) or water (red line); b) Double bond conversion of P-Lig prepared in ionic liquid (blue line) or water (red line) (Inset: Digital images of the photocuring process of P-Lig prepared in water).

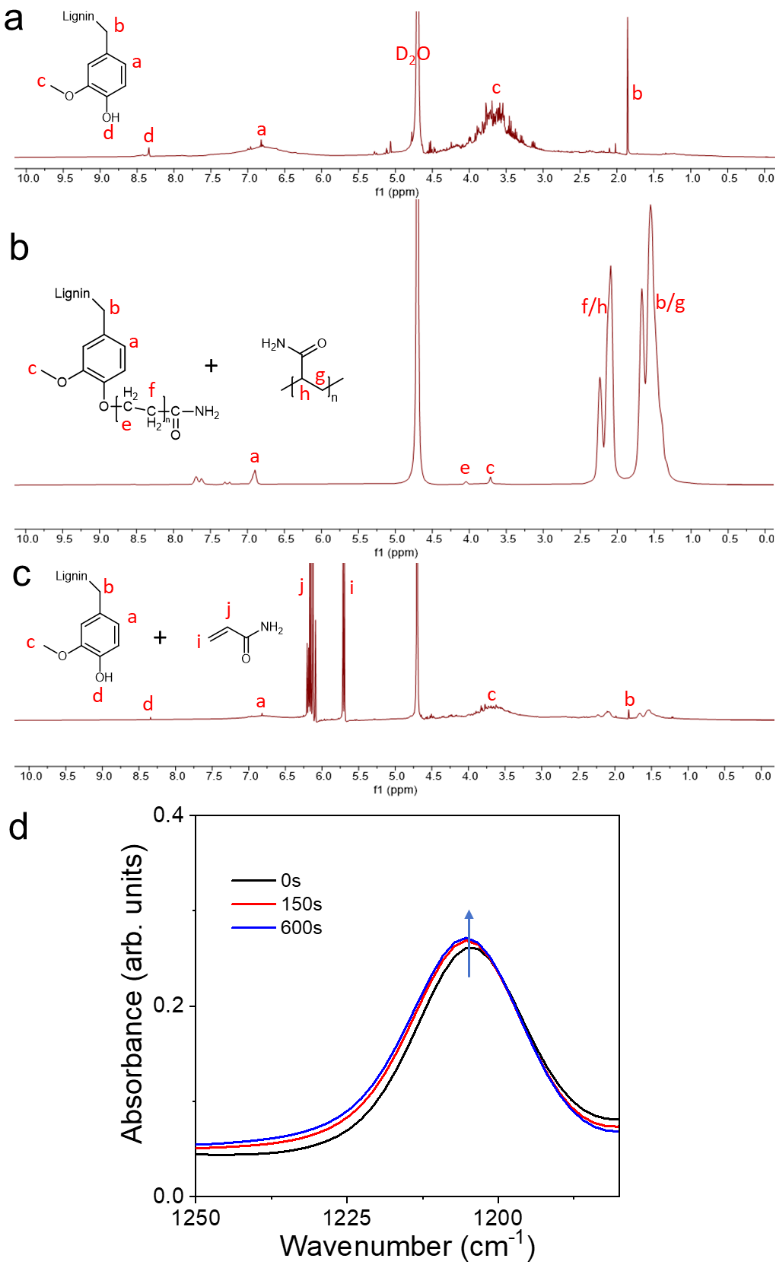

Supplementary Fig. 9 The interaction between lignosulfonate and acrylamide in P-Lig. ^{1}H NMR spectrum of a) lignosulfonate, b) the products of the reaction between lignosulfonate and acrylamide in ionic liquid upon UV irradiation, and c) physical mixture of lignosulfonate and acrylamide without light irradiation; d) In situ FT-IR spectra of P-Lig upon UV irradiation for 0 s (black line), 150 s (red line) and 600 s (blue

line).

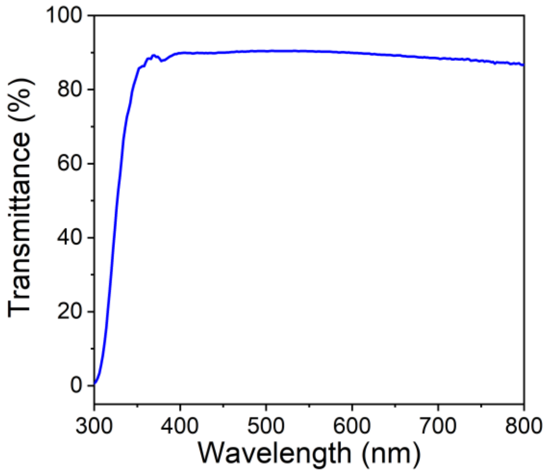

Supplementary Fig. 10 UV-vis transmittance spectra of the P-Lig (the thickness of P-Lig is 1 mm).

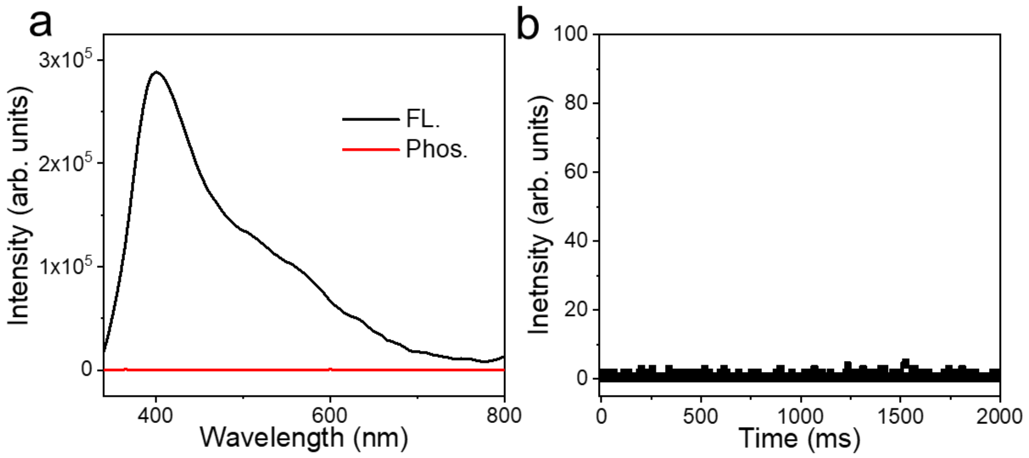

Supplementary Fig. 11 RTP emission of lignosulfonate in ionic liquid. a) Standard (black line) and delayed (red line) emission spectra of lignosulfonate dissolved in ionic liquid; b) Phosphorescent lifetime of the lignosulfonate dissolved in ionic liquid (no signal detected). In all cases $\lambda_{exc.}$= 320 nm, $\lambda_{collected}$= 510 nm, delay time = 10 ms.

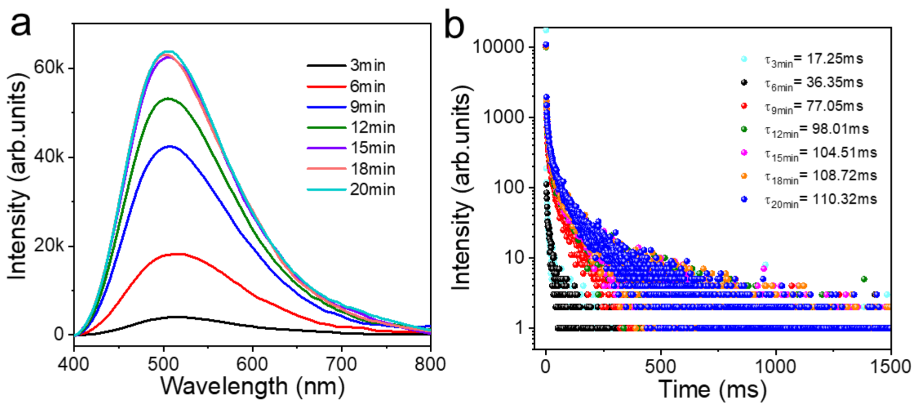

Supplementary Fig. 12 RTP intensity and lifetime of P-Lig during the photocuring process. a) RTP Intensity; b) RTP Lifetime. In all cases $\lambda_{exc.}$= 320 nm, $\lambda_{collected}$= 510 nm, delay time = 10 ms.

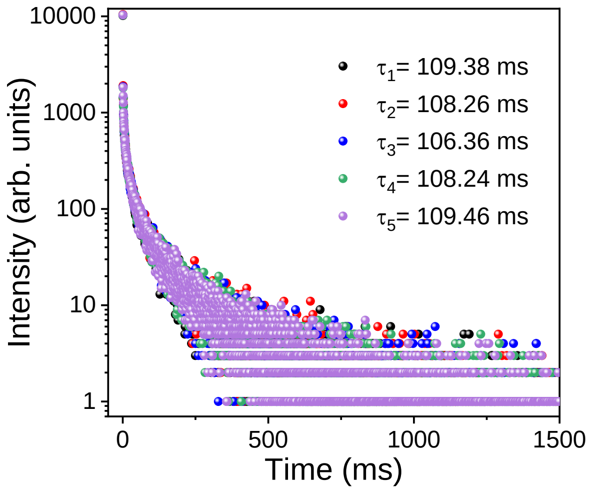

Supplementary Fig. 13 Phosphorescent lifetime of P-Lig that reproduced using the method for 5 times. In all cases $\lambda_{exc.}$= 320 nm, $\lambda_{collected}$= 510 nm, delay time = 10 ms.

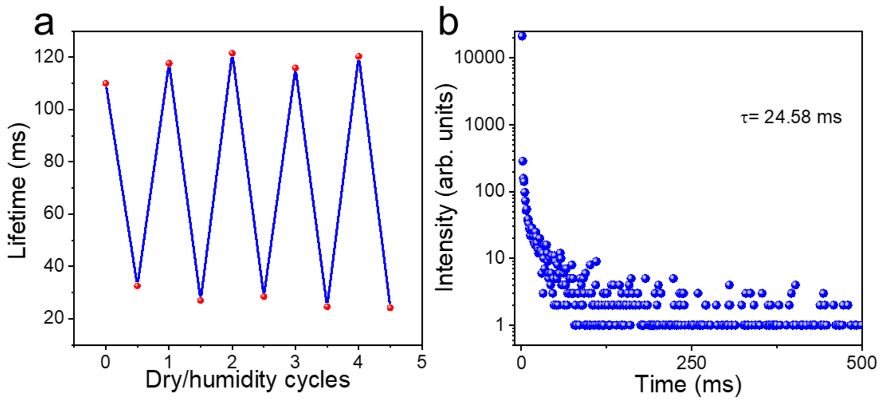

Supplementary Fig. 14 RTP performance of P-Lig upon recycling of humidity and drying cycles. a) RTP lifetime of P-Lig upon recycling of humidity (80 %) and drying (80 °C) cycles; b) Phosphorescent lifetime of P-Lig upon humidity treatment.

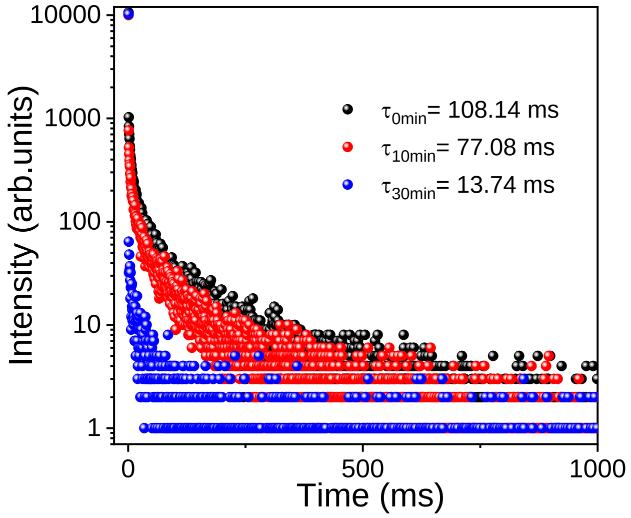

Supplementary Fig. 15 Phosphorescent lifetime of P-Lig after immersing in water for different times. In all cases $\lambda_{exc.}$= 320 nm, $\lambda_{collected}$= 510 nm, delay time = 10 ms.

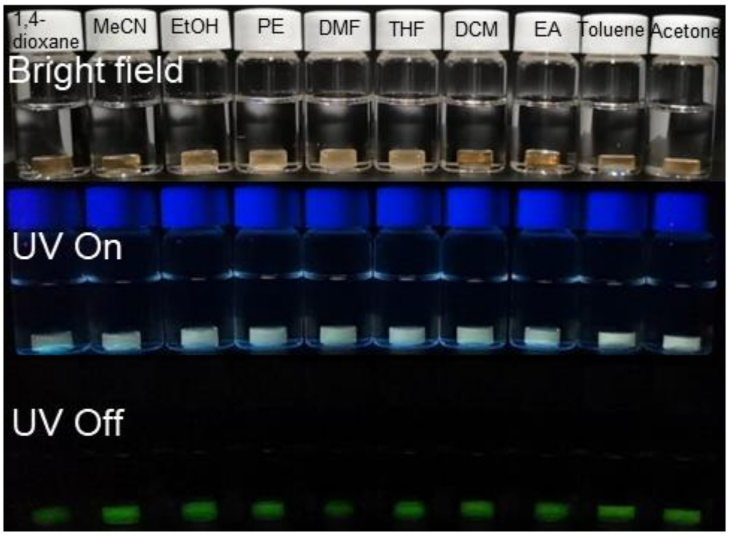

Supplementary Fig. 16 Digital images of P-Lig that were immersed in different solvents for three months.

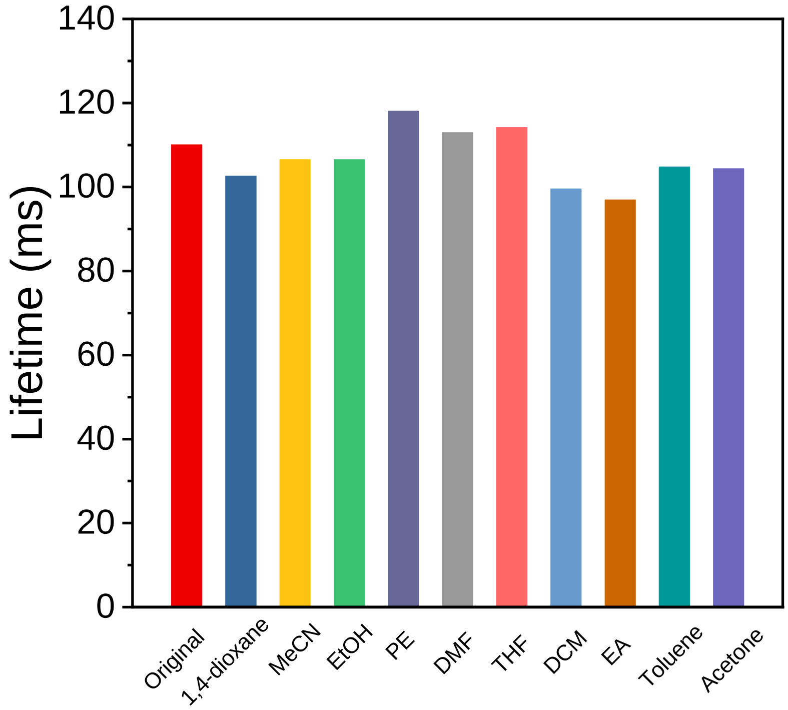

Supplementary Fig. 17 Phosphorescence lifetime of P-Lig that were immersed in different solvents for three months. In all cases $\lambda_{exc.}$= 320 nm, $\lambda_{collected}$= 510 nm, delay time = 10 ms.

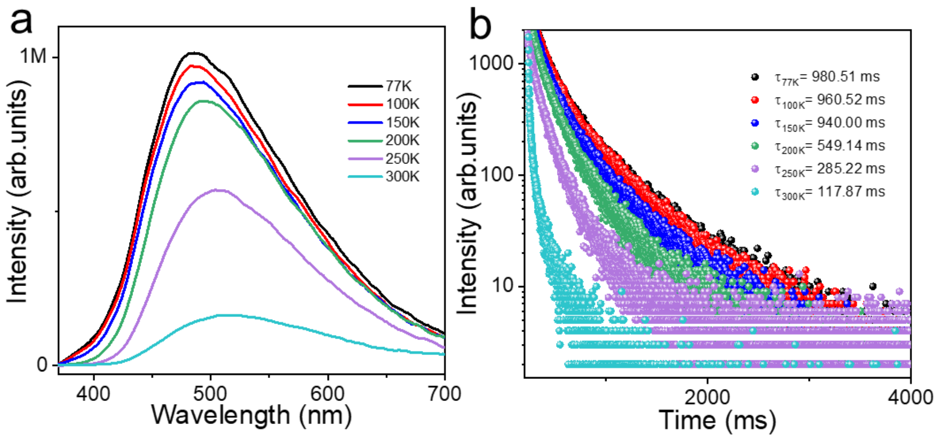

Supplementary Fig. 18 Temperature dependent a) Phosphorescence spectra and b) Lifetime of P-Lig ($\lambda_{exc.}$= 320 nm, $\lambda_{collected}$ = 510 nm).

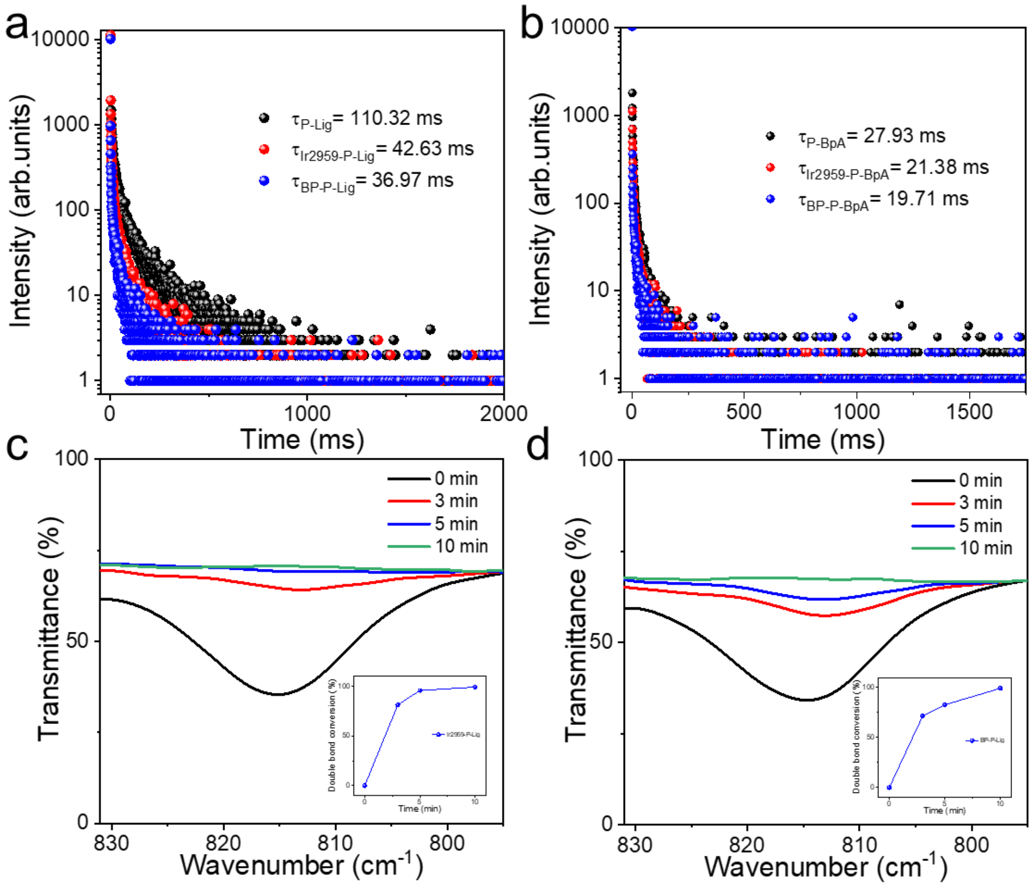

Supplementary Fig. 19 RTP lifetime and double bond conversion of samples that prepared by different photoinitiators and chromophores. a) Phosphorescence lifetime of P-Lig (lignosulfonate as the chromophore) initiated by lignosulfonate (black), Ir2959 (red) and benzophenone (blue); b) Phosphorescence lifetime of P-BpA (phenylboronic acid as the chromophore) initiated by ammonium persulphate (black), Ir2959 (red) and benzophenone (blue); c) FT-IR spectra of P-Lig initiated by Ir2959 (Inset: Double bond conversion of Ir2959-P-Lig); d) FT-IR spectra of P-Lig initiated by benzophenone (Inset: Double bond conversion of BP-P-Lig). In all cases $\lambda_{exc.}$= 320 nm, $\lambda_{collected}$= 510 nm, delay time = 10 ms.

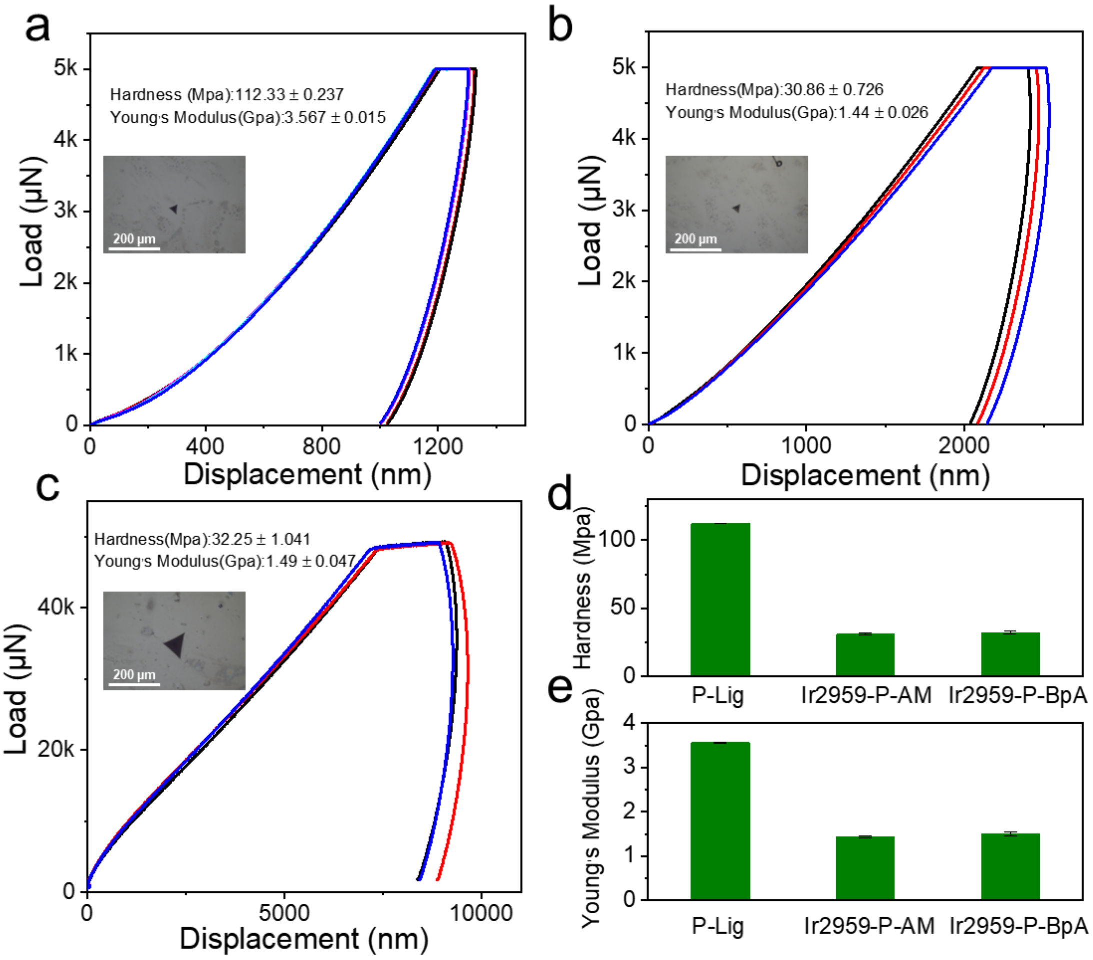

Supplementary Fig. 20 Mechanical properties of P-Lig, Ir2959-P-AM and Ir2959-P-BpA. Nanoindentation tests of a) P-Lig, b) Ir2959-P-AM, c) Ir2959-P-BpA (Scale bar of the inset was 200 μm); Comparison of d) Hardness and e) Young's modulus of the mentioned above three materials (Error bars indicate the standard deviations for three separate measurements of the samples, the value for hardness and young's modulus of P-Lig are 112.33 ± 0.237 Mpa and 3.567 ± 0.015 Gpa, respectively; the value for hardness and young's modulus of Ir2959-P-AM are 30.86 ± 0.726 Mpa and 1.44 ± 0.026 Gpa, respectively; the value for hardness and young's modulus of Ir2959-P-BpA are 32.25 ± 1.041 Mpa and 1.49 ± 0.047Gpa, respectively).

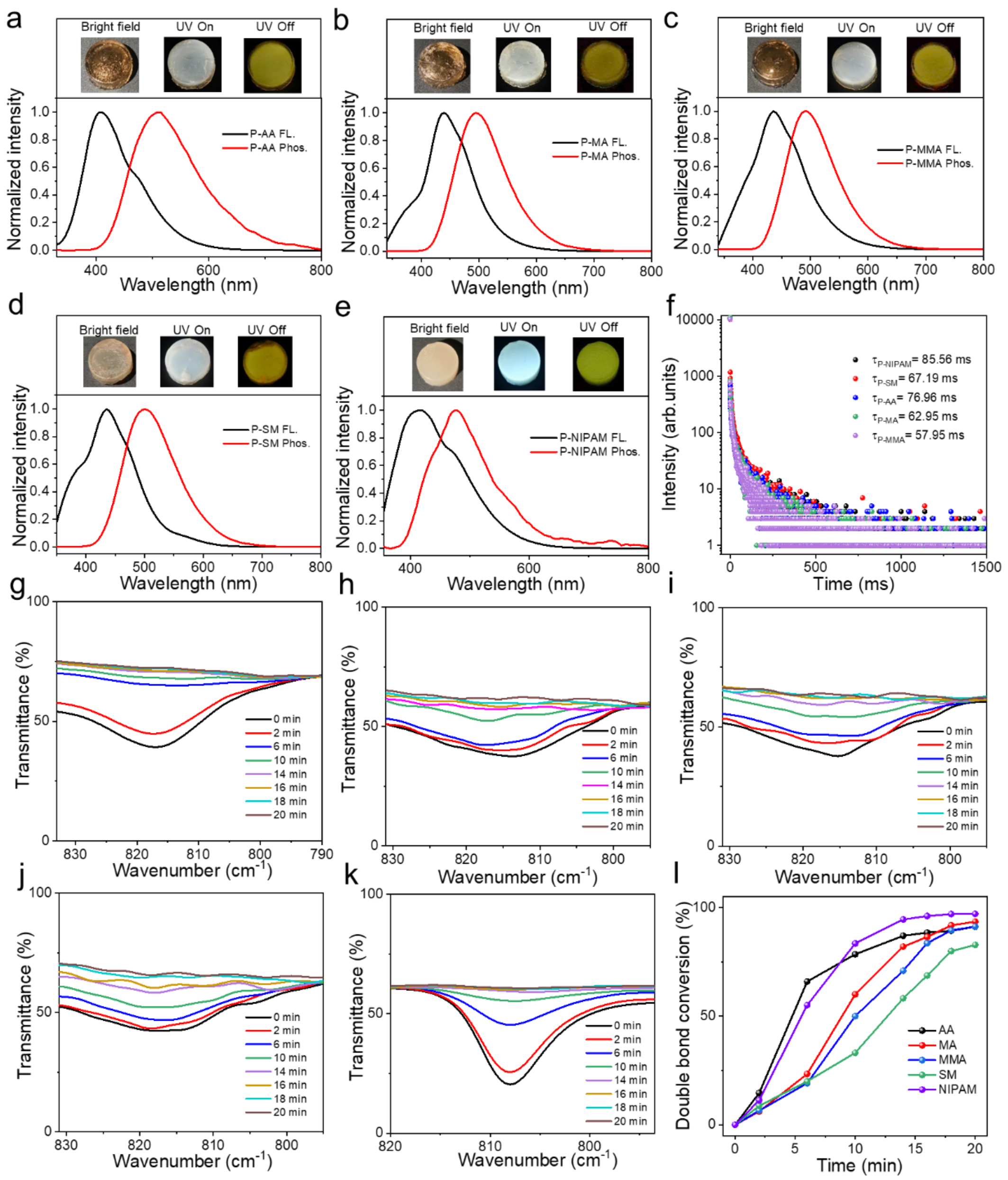

Supplementary Fig. 21 Emission spectra and double bond conversion of P-Lig prepared using different monomers. Standard (black line) and delayed (red line) emission spectra of a) P-AA ($\lambda_{exc.}$= 310 nm, $\lambda_{collected}$= 510 nm), b) P-MA ($\lambda_{exc.}$= 320 nm, $\lambda_{collected}$= 510 nm), c) P-MMA ($\lambda_{exc.}$= 320 nm, $\lambda_{collected}$= 510 nm), d) P-SM ($\lambda_{exc.}$= 330 nm, $\lambda_{collected}$= 500 nm), e) P-NIPAM ($\lambda_{exc.}$= 310 nm, $\lambda_{collected}$= 490 nm), Inset: the images of polymers in daylight (left), polymers upon excitation by UV light source (middle) and polymers after switching off the UV light source (right); f) Phosphorescence lifetime of the polymers mentioned above at the corresponding collected emission wavelength; g) - k) FT-IR spectra of the photocured materials used different monomers

in this system upon UV irradiation for different times (acrylic acid, methyl acrylate, methyl methacrylate, styrene and N-isopropylacrylamide); l) Conversion of double bonds of the materials mentioned above upon UV irradiation for different times. In all cases delay time is 10 ms.

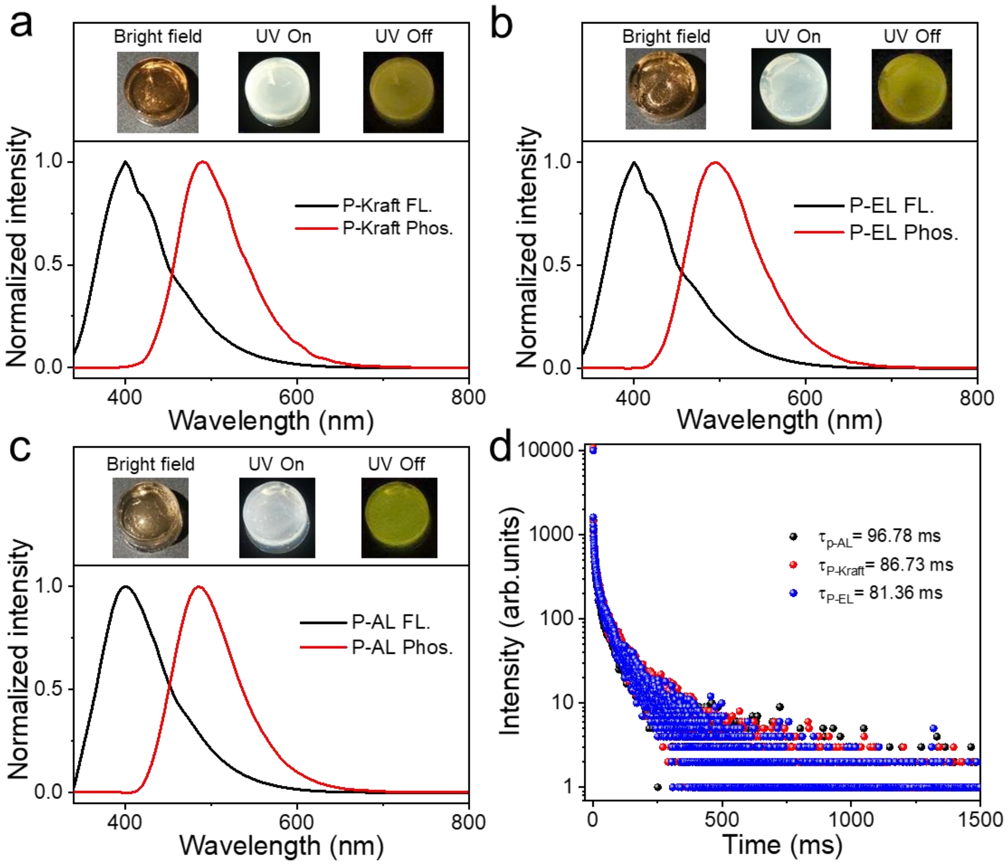

Supplementary Fig. 22 RTP spectra and lifetime of P-Lig prepared by different kinds of lignin. Standard (black line) and delayed (red line) emission spectra of a) P-Kraft (Kraft lignin), b) P-EL (Enzymatic hydrolysis lignin), c) P-AL (alkali lignin), Inset: the images of polymers in daylight (left), polymers upon excitation by UV light source (middle) and polymers after switching off the UV light source (right); d) Phosphorescence lifetime of the polymers mentioned above. In all cases $\lambda_{exc.}$= 320 nm, $\lambda_{collected}$= 510 nm, delay time = 10 ms.

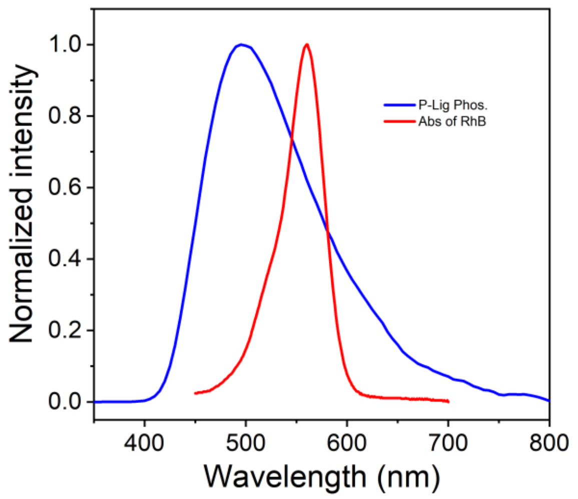

Supplementary Fig. 23 Phosphorescence emission of P-Lig (blue line) and absorbance of RhB (red line) that were dissolved in ionic liquid.

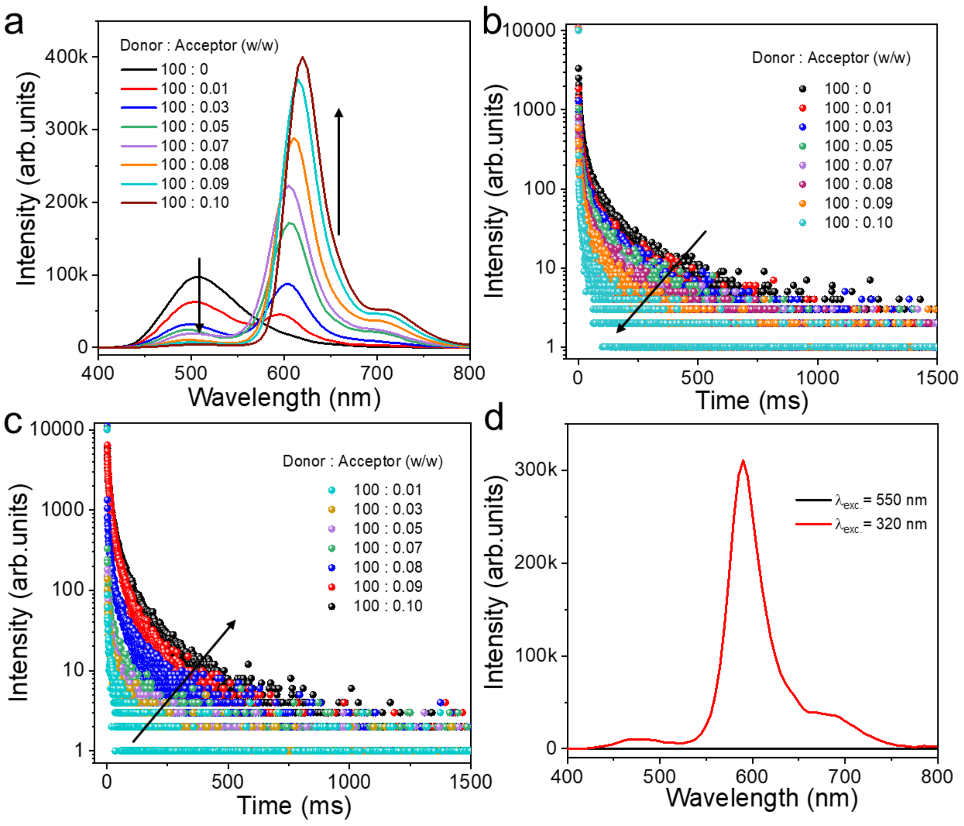

Supplementary Fig. 24 RTP emission and lifetime of P-Lig/RhB with different RhB loading contents. a) Delayed emission spectra of P-Lig/RhB ($\lambda_{exc.}$= 320 nm); b) Lifetime decay plots of P-Lig/RhB ($\lambda_{collected}$= 510 nm) and c) Lifetime decay plots of P-Lig/RhB ($\lambda_{collected}$= 600 nm) with increasing the doping concentration of RhB; d) Delayed emission spectra of P-Lig/RhB (100:0.07) upon direct excitation ($\lambda_{exc.}$= 550 nm, black line) and upon indirect excitation ($\lambda_{exc.}$= 320 nm, red line), the delay time

was 10 ms.

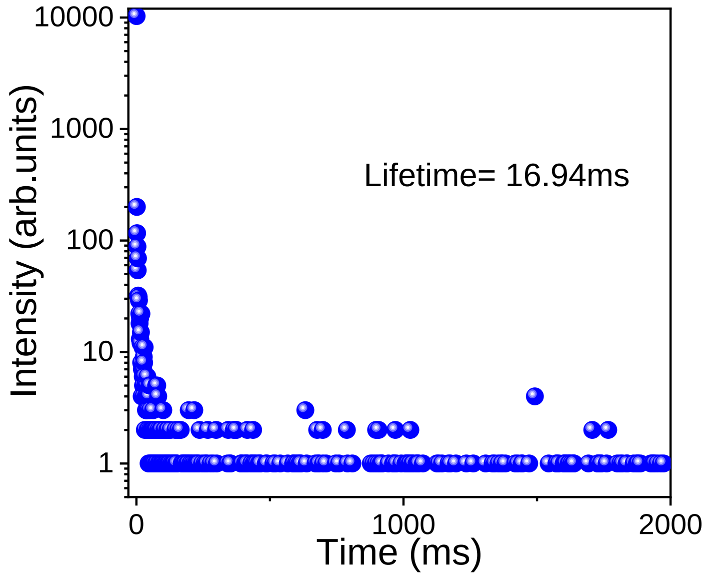

Supplementary Fig. 25 Phosphorescence lifetime of the sample P-AM that was initiated by thermal initiator (Ammonium persulfate) ($\lambda_{exc.}$= 320 nm, $\lambda_{collected}$= 510 nm, delay time = 10 ms).

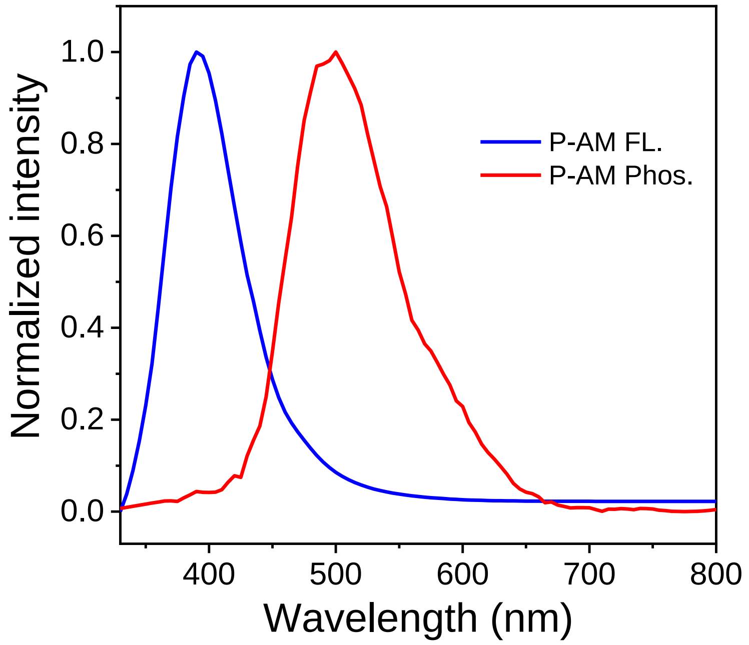

Supplementary Fig. 26 Standard (blue line) and delayed (red line) emission spectra of P-AM initiated by ammonium persulfate ($\lambda_{exc.}$= 320 nm, $\lambda_{collected}$= 510 nm, delay time = 10 ms).

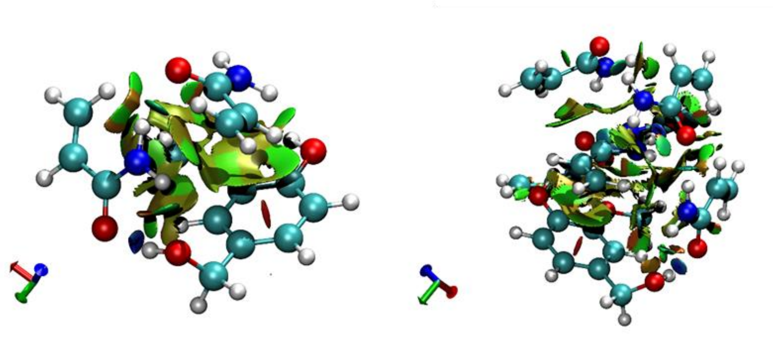

Supplementary Fig. 27 Calculated interaction model between lignosulfonate and different numbers of acrylamide monomers (Left - lignin with two monomers, the value was -10.39 eV and right - lignin with six monomers, the value was -13.82 eV).

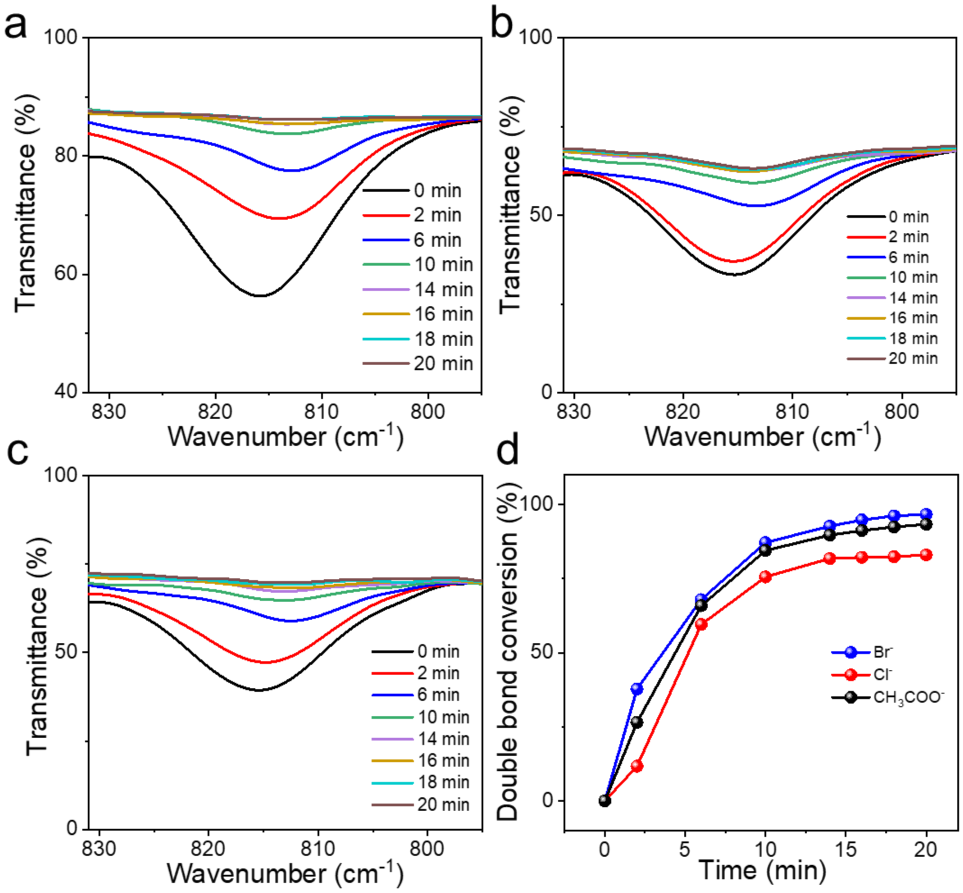

Supplementary Fig. 28 Double bond conversion of P-Lig prepared using different ionic liquids. a) – c) FT-IR spectra of the photocured materials using different ionic liquids in this system upon UV irradiation for different times (1-ethyl-3-methylimidazolium bromide, 1-ethyl-3-methylimidazolium chloride, 1-ethyl-3-methylimidazolium acetate); d) Conversion of double bonds of the materials mentioned above upon UV irradiation for different times.

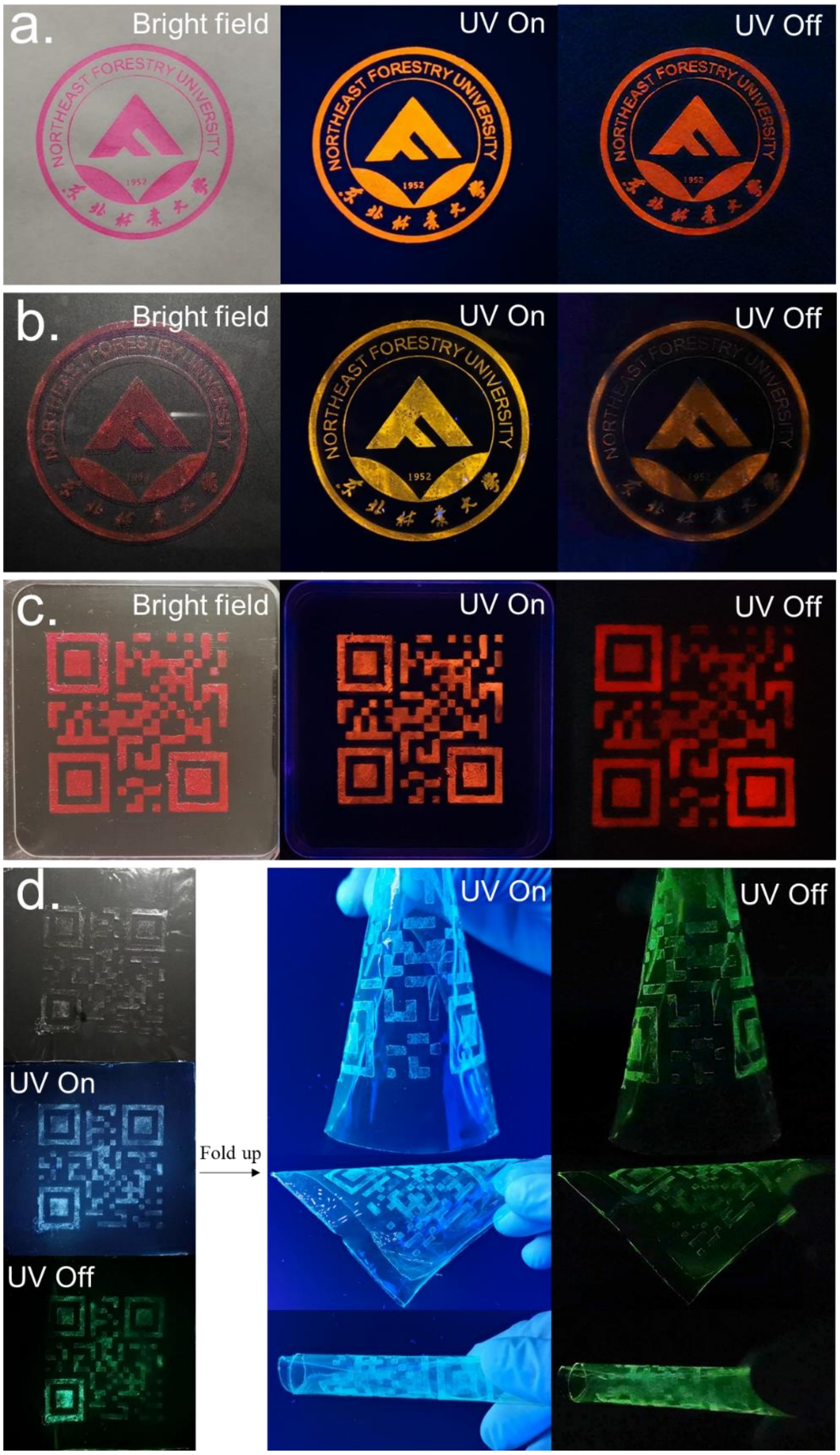

Supplementary Fig.29 Digital photographs of P-Lig or P-Lig/RhB printed on different substrates. a) School badge on paper; b) School badge on glass; c) QR code on plastic (Polystyrene); d) QR code (P-Lig coating) on foldable polyvinyl alcohol.

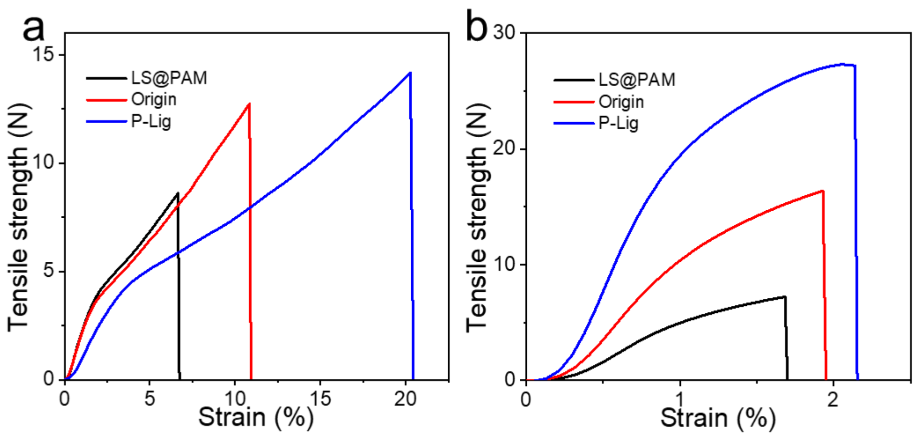

Supplementary Fig. 30 Mechanical properties of yarns treated in different ways. a) Tensile strength and strain of original yarns (red line), yarns treated by lignosulfonate -derived RTP materials prepared in the previous reports (black line) and yarns treated by the photocured RTP materials in this work (blue line); b) Tensile strength and strain of original papers (red line), papers treated by lignosulfonate -derived RTP materials prepared in the previous reports (black line) and yarns treated by the photocured RTP materials in this work (blue line).

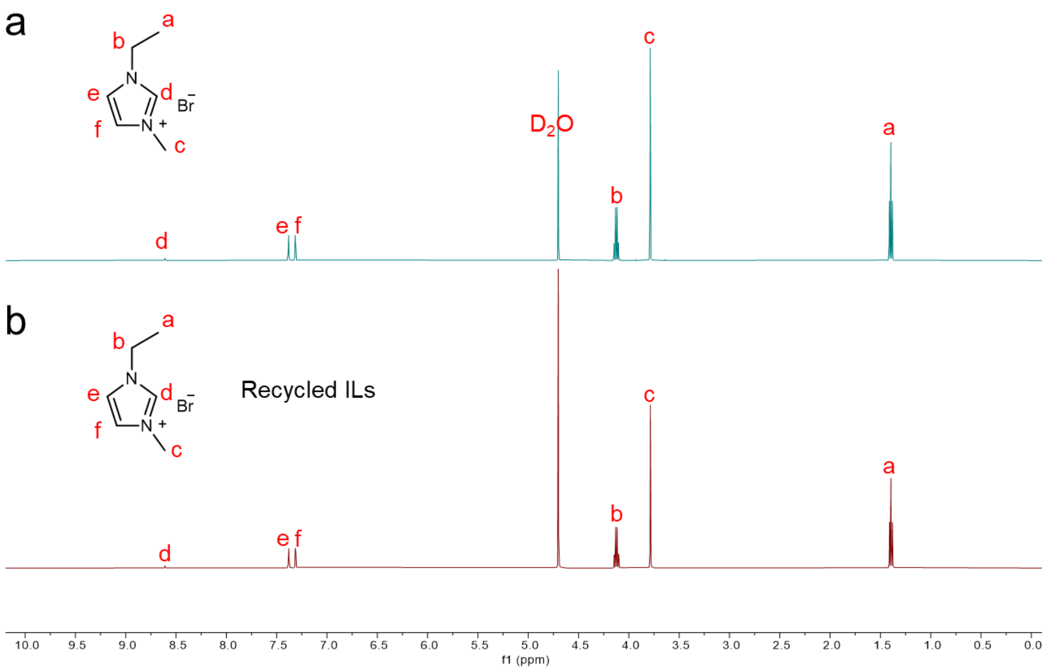

Supplementary Fig. 31 ^{1}H NMR spectrum of a) 1-ethyl-3-methylimidazolium bromide purchased from Aladdin (Shanghai, China), and b) Recycled ionic liquid from P-Lig.

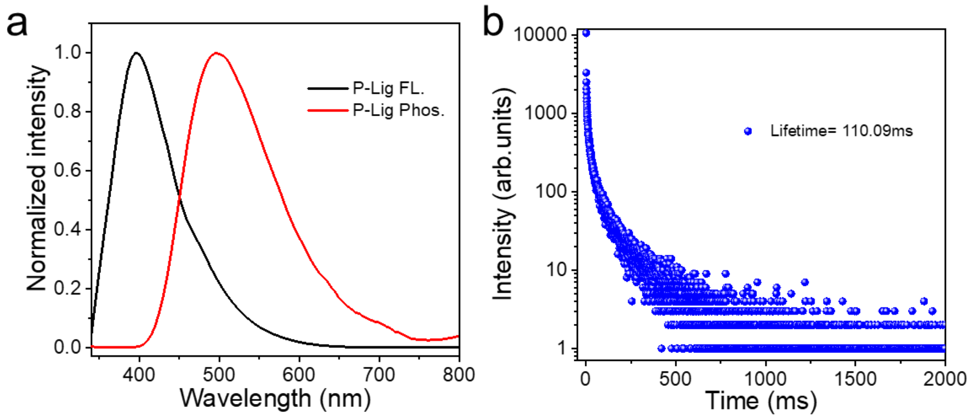

Supplementary Fig. 32 Emission spectra and RTP lifetime of P-Lig prepared using recycled ionic liquid. a) Standard (black line) and delayed (red line) emission spectra of P-Lig prepared using the recycled ionic liquid; b) Phosphorescence lifetime of the P-Lig mentioned above ($\lambda_{exc.}$= 320 nm, $\lambda_{collected}$= 510 nm, delay time = 10 ms).

Supplementary Table 1 Summary of energy transfer (Φ_{et}) efficiency.

Acceptor	Donor(**P-Lig**) and Acceptor (**RhB**) Ratio (w/w)	Average Lifetime (in ms) of **P-Lig** at 510 nm ($\lambda_{exc.}$= 320 nm)	Energy Transfer Efficiency (%)
**RhB**	100:0	110.32	-
**RhB**	100:0.01	92.09	16.5
**RhB**	100:0.03	88.89	19.4
**RhB**	100:0.05	83.91	23.9
**RhB**	100:0.07	72.78	34.0
**RhB**	100:0.08	59.67	45.9
**RhB**	100:0.09	39.08	64.6
**RhB**	100:0.10	22.34	79.7

Supplementary References

1. Grimme S, Antony J, Ehrlich S, Krieg H. A consistent and accurate ab initio parametrization of density functional dispersion correction (DFT-D) for the 94 elements H-Pu. *J. Chem. Phys.* **132,** 154104 (2010).
2. Neese F. Software update: the ORCA program system, version 4.0. *WIREs Comput. Mol. Sci.* **8,** e1327 (2018).
3. Grimme S, Ehrlich S, Goerigk L. Effect of the damping function in dispersion corrected density functional theory. *J. Comput. Chem.* **32,** 1456-1465 (2011).
4. Marenich AV, Cramer CJ, Truhlar DG. Universal Solvation Model Based on Solute Electron Density and on a Continuum Model of the Solvent Defined by the Bulk Dielectric Constant and Atomic Surface Tensions. *J. Phys. Chem. B*. **113,** 6378-6396 (2009).
5. Johnson ER, Keinan S, Mori-Sánchez P, Contreras-García J, Cohen AJ, Yang W. Revealing Noncovalent Interactions. *J. Am. Chem. Soc.* **132,** 6498-6506 (2010).
6. Lu T, Chen F. Multiwfn: A multifunctional wavefunction analyzer. *J. Comput. Chem.* **33,** 580-592 (2012).
