# Supplementary material for: Photocured room temperature phosphorescent materials from lignosulfonate
Source: Nat Commun. 2024 Feb 21;15:1590. doi: 10.1038/s41467-024-45622-3 (PMC10881523; doi:10.1038/s41467-024-45622-3)
Supplement: Supplementary file 3 — Description of Additional Supplementary Files [file 41467_2024_45622_MOESM3_ESM.docx]

Title: Supplementary Movie 1

Description: The afterglow video of the 3D bulk material made from P-Lig and P-Lig/RhB
